# Extracts

**Published:** 1881-12

**Authors:** 


### Extracts.

Recent Progress in Peritoneal Surgery.—In a paper read before the New York Academy of Medicine on the above subject (*Boston Med. and Surg. Journal*), Dr. J. Marion Sims gives expression to the following conclusions:

(1.) Wounds of the peritoneal cavity, however produced, have to run a common course.

(2.) They have a common termination in death, and that death is by septicæmia.

(3.) This is the general law in deaths from ovariotomy.

(4.) This is the general law after gun-shot and other wounds of the abdomen.

(5.) The septicæmia is the result of the absorption of bloody serum remaining in the peritoneal cavity after wounds or operations.

(6.) In gun-shot wounds of the pelvic cavity the tendency is to recovery, on account of the natural drainage in this situation.

(7.) Gun-shot wounds of the abdominal cavity prove fatal from septicæmia, because there is no natural drainage, and the bloody serum poured out becomes absorbed.

(8.) In order to prevent this it is necessary to open the abdomen, clear out the peritoneal cavity, tie the bleeding vessels, suture the intestines or other tissues requiring it, and possibly insert a drainage tube.

(9.) If the operation is properly performed, it is rarely necessary to make use of a drainage tube.

Dr. Sayre being called upon by the chair to open the discussion when Dr. Sims concluded, remarked that it was, perhaps, the most able paper to which he had ever listened. The author of it had covered the whole ground, and there was but little left to be said upon the subject, but he could not refrain from endorsing Dr. Sims' position as to the vital importance of drainage. Why was it that patients recovered who received pelvic wounds? For the simple reason that they were through and through wounds, and an opportunity was thus afforded for successful drainage.

Dr. Fordyce Baker, the President of the Academy, stated that the special aim of the paper was to show the importance of general surgeons availing themselves of the advances that had recently been made by gynæcological surgeons, and called upon Professor James R. Wood to speak upon the subject.

Dr. Wood said that while he felt very grateful to Dr. Sims for his great paper, he believed that there were things in it which nobody else in the world would dare to say, and he would therefore be loath to express an opinion indorsing all the points that had been maintained by the distinguished writer. In general surgery he considered it very dangerous to invade the abdomen unless an accurate diagnosis was possible in every case where it was attempted. He had now had a very extensive experience in regard to this subject, and he could not but confess that in his earlier days he had sometimes tried to find balls in the abdomen which he ought not to have looked for. As to the bloody serum upon which so much stress had been laid, he was disposed to think that this was often a post-mortem phenomenon, and in many cases of autopsies after fatal septicæmia he had failed to find it altogether. In wounds of the liver there was usually very free hemorrhage, and it would be impossible to control this, even if the abdomen were opened. In these cases the patient generally died within six or eight hours after the reception of the injury, and if the surgeon cut into the abdomen at all it had to be done before the patient had recovered from the shock. The chances were that death would take place while he was engaged in cleaning out the peritoneal cavity, and this would not be a very good thing for the operator's reputation. So, if in a case of laceration of the stomach, the surgeon attempted to sew up the organ he would get into trouble, because, as a rule, such patients died of shock; and did not live long enough to get septicæmia. In ovariotomy the case was very different, for there the surgeon could choose his own time for operating, and have his patient all prepared; but in abdominal wounds the trouble was that this terrible shock stared him constantly in the face.

---

Ophthalmia Neonatorum.—The disease usually shows itself in from the second to the sixth day after birth. At this time there will be observed in the eye of the child, after a nap, some gluing together of the lids or an accumulation of mucus or muco-pus at the inner canthus, and probably in a few hours a slight edema of the lids. This edema may precede the discharge of matter.

As to treatment, I will first speak of that relating to the aborting of the disease. When it is known that there is an existing leucorrhœa or gonorrhœa, of course the risk of contagion can be lessened by great cleanliness upon the part of the mother, in connection with certain medicated vaginal injections. Immediately after the birth of the child the parts around the eyes should be thoroughly washed. If there is any doubt about the efficacy of the washing, a three or five-grain solution of nitrate of silver may be dropped into the eyes and allowed to remain there a minute, when it should be well washed out with a solution of common salt (a teaspoonful to the half pint of water). If there is much reaction from the drops, it can soon be allayed by the application of cold cloths. If the disease has gained a foothold, the first indication is cleanliness. The eyes should be thoroughly washed every two hours. The cleansing should be done by means of small bits of cloth. No cleansing material which cannot be thrown away immediately after use should be employed. The sponge, which from its texture cannot be readily cleansed, and so may serve as a carrier of contagion, is bad enough, but the syringe is far worse. This instrument may not only serve as a medium of accidental reinoculation, but from application of undue force the fluid may strike the diseased eye, and, returning contaminated with the purulent secretion, find its way into the eye of the surgeon or attendant, where it is almost certain to reproduce the disease. This accident has several times occurred under my observation. Again, the tip of the syringe may puncture the cornea and lead to destructive intraocular inflammation. This accident may appear to be the result of culpable carelessness, but I have known it several times to occur under the hands of careful men. Bits of absorbent cotton or soft old cloths will be found by far the best material for cleansing purposes.

It may be asked how the matter is to be removed from the cul-de-sac. This may be easily accomplished by gentle manipulation of the lids, or after a method which I will presently describe. After cleansing the eye medicated applications should be resorted to. On being called to a case of ophthalmia neonatorum it is my custom to first order tannic acid (five-grain solution), to be used in the following manner: Immediately after cleansing the eye, as before directed, one or two drops of this solution must be put into the eye and allowed to remain for a minute or two. Opening the eye at the end of this time, it will be observed that the matter has coagulated, and can now be easily removed in strings or shreds. Removing these by means of a little absorbent cotton twisted upon a match, the same solution is again dropped into the eye, and the coagulum removed as before.

Tannic acid does not serve in all cases, and when a substitute is required nitrate of silver (one to fifteen grains to the ounce of distilled water) will best serve the purpose. This is to be used in the same manner as the tannic acid, with the extra precaution that it must be washed out of the eye almost immediately with a solution of common salt.. This does away with all possibility of silver staining. Some physicians have an unreasonable dread of nitrate of silver as a means of treatment in diseases of the eye. I believe that next to tannic acid it is the best agent for application in almost every disease of the lids. For aborting gonorrhœal or purulent ophthalmias no other drug can equal it, for it destroys all germs of contagion. I no more hesitate to employ it than I do sulphate of atropia, when I can myself make the applications; and with due care a case of silver-staining will, I am confident never occur.

Edema of the lids may be rapidly reduced by the application of iced cloths. Previous to this application the lids should be well annointed with either simple cerate or some other unctious substance, that the delicate skin of the infant may be protected from the constant moisture. If this precaution is neglected excoriation is likely to occur. In mild cases, or in certain stages of the more severe ones, the common astringents—such as sulphate of zinc, alum, and borax—will sometimes be of service.

Atropia sulphate or eserine sulphate will be often called for, either to prevent suppuration of the cornea or, where this has already begun, to stay its progress or hasten its cure.

Although operative procedures—such as paracentesis of the cornea when ulcerative keratitis occurs, or canthodectomy when there is dangerous pressure of the swelled lids upon the globe—are sometimes called into requisition, I have never found these operations necessary.

One therapeutic agent which should be scrupulously avoided in the management of this disease is the poultice.

Ophthalmia neonatorum, though always serious, and in the light of statistics the most fruitful source of blindness, is a disease which admits of comparatively easy control; and experience fully warrants the assertion that when seen in its earlier stages, and promptly submitted to careful and intelligent treatment, it need never result in the loss of the eye.—*W. Cheatham, M.D., in Louisville Med. News.*

---

THERAPEUTIC NIHILISM.—The recognition of ill-fed muscles from impairment in the power of assimilating albuminoids, as a condition involving the heart and diaphragm, is a matter which must engage the attention of the profession in a little time; indeed, when it is sated with nerve pathology and poison-germs, and can turn its attention to something else. . . . . .

If the medical man speak to the patient with doubtful accents and hesitating utterances, he does not inspire confidence; he really sows distrust. This is the explanation of the successful treatment of a case by one man, where another has failed; the remedial measures being much the same. The one carries the patient with him to the restoration of health; the other intensifies a morbid state and tends to make it permanent.

This is a matter too little thought about. Just as a weak-willed medical man fails to do certain patients good; and lack of decision of character unfits a medical man for dealing with emergencies, where the judgment must be prompt and the action energetic; so the thereapeutic nihilist, who doubts the efficacy of drugs and leaves the patient to nature, disheartens many patients, and leaves them chronic valetudinarians; while in the hands of an enthusiast the cases would soon move onward to a satisfactory termination. There are some men who are "doubting Thomases;" there are others who decry what they do not understand, and deprecate remedies with whose potency they are unacquainted, who do infinite, immeasurable harm to their patients. An eclipse of faith in medicines has now existed some time; but the darkness is beginning to move away, and a return of faith, stronger, firmer, more capable of giving a *raison d'etre* for its existence than in the past is dawning — the daybreak of happier times for those who are stricken down with illness, or crippled in their working power by incapacity in their digestive viscera. This therapeutic nihilism is a passing wave of opinion, a temporary mental state, the end of which is at hand; and the sooner it is over the better for all. The patient's prospects will be all the brighter, the medical man all the happier for feeling that the patient has got some "value received" in return for his outlay. A healthier condition of thought on matters medical will generally obtain; for quacks, charlatans and irregular practitioners of all kinds are to a great extent fostered by the recent want of faith in the medical profession. When a man is sick, what he wishes is to get well.; the means to him is a matter of comparative indifference. . . . . . . . . . .

Perhaps those acrid personages who have a distinct line of faith, or rather the want of it, those individuals who believe in Homœopathy, and talk flippantly of "Allopaths," who deny the utility of the contagious disease act, who are anti-vaccinators, who are blinded compounds of sceptcism and credulousness; those who are utterly unteachable from prejudice and ignorance "vaunting itself as knowledge" cannot be benefited; not even if their chief diurnal instructor, their "guide, philosopher and friend," the *Daily News*, was to modify its attitude and show a livelier interest in matters affecting the public health, and a little more decent respect for the observations of "the natural man." They are an unhopeful class, the obstructionists of all progress in matters sanitary and hygienic; whose self-satisfaction in their ignorance on matters medical is simply as aggressive and impertinent as that ignorance is appalling. They are blinded guides in their self-appointed mission of directing the opinions of mankind: but their faith in themselves is unbounded! *Fothergill's Indigestion and Biliousness.*

A Method of Curing Hydrocele without Confinement of the Patient for Half an Hour During the Treatment.—In the third year of our late war, an ordnance officer complained of swelling of the left testicle, which only annoyed him from its size. Upon examination, it was found to be an ordinary hydrocele of medium size. I explained to him the ordinary operation of tapping and injecting, and that it would require him to lay up about a week, or perhaps longer, in fact, until the swelling of the testicle produced by the injection had subsided. This confinement he thought it impossible for him to undergo, and begged me to try something which would not require him to lay up.

On the 20th of April, 1864, without drawing off the water of the tumor, I injected, with a hypodermic syringe, about thirty drops of strong comp. tincture of iodine; thinking that the dilution of the iodine in the fluid of the hydrocele would stimulate the sac sufficiently and that the next day the water could be drawn off and the surfaces of the vaginal sac be thus allowed to come in contact. To my surprise, the next day, the hydrocele was not half the size; the fluid had been absorbed. Instead, therefore, of drawing off the water, on the 23rd I repeated the iodine injection, and on the 26th, though the swelling had been still more reduced, I again threw in the iodine. On the 30th, the fluid had disappeared, though the vaginal coverings and the testicle itself were thicker and hung down lower than on the side not implicated. He wore a suspensory bag from the third day after the first injection and I directed him to continue to wear this, making it a little tighter than he had been wearing it. From the first injection, this patient experienced no pain or inconvenience and did not lose an hour from his work.

He had no return of his disease six months after the operation: the cure was therefore complete.

Encouraged by the success of this operation I have treated successfully eleven other cases, and my friend Dr. I. S. Mitchell, at my suggestion, has treated five cases with like success. I have not tried this in very old hydroceles. I doubt if it would succeed in such. In these cases I have first evacuated the water, and then injected ʒi strong tincture of iodine and left it in the sac, and applied a tight suspensory bandage; the pain and swelling have been severe, but the cures have been eventually good. If, then, this troublesome and not uncommon disease may be cured by the above simple operation, without the patient losing an hour from his ordinary business, as these cases would show, it would be an improvement in surgical treatment of such cases to adopt this operation, instead of the old plan of tapping and injecting iodine, port wine, sulphate of zinc, etc.—*T. L. Ogier, M.D., in Gaillard's Medical Journal.*

---

THE POISONOUS PROPERTIES OF QUININE.—Quinine is unquestionably at times a powerful nervine irritant. I have often had occasion to deplore its effects upon the brain, especially in infants and young children, when, after several days of persistent use, it had completely upset the nervous system, producing great wakefulness, restlessness and fright, with an alarming sense of falling, ending some times in convulsions and even in death. I have become so cautious in the use of this remedy of late years that I have had no occasion to witness its poisonous effects in my own practice, but, in consultation, I have had opportunities of confirming the views which I have so long entertained as to its immediate and direct agency in producing the effects which I have ascribed to it.

I am under the impression that the revival of its use in the large doses in which it was given at the South thirty or forty years ago is doing a great deal of mischief throughout the country, and especially in some of our larger cities, and that it is often the cause of fatal injury when its agency is not suspected.—*William O. Baldwin, M.D., in Medical Gazette.*

---

QUININE POISONING.—The publication of Dr. Baldwin's paper on the Poisonous Properties of Quinine in Large Doses in 1847, served the good purpose of bringing many of us at the South to think seriously of its dangers under certain conditions. In the main we profited by his teachings, and came down to a common sense view of the subject. We at once stopped giving 60, 80 and 100 grains of quinine during the remission of fever and reduced the quantity to from 24 to 36 gr. in the 24 hours, or just enongh to produce moderate cinchonism. Dr. Baldwin was greatly aided in the revolution he effected in this direction at the South by several accidents proving positively the dangers of quinine in heroic doses. One of them I may mention: A lady aged 30, had an attack of pernicious intermittent or as we used to term it, congestive fever. She was seen by one of our most eminent physicians, a man whose contributions to medical literature stamp him as one of the ablest men the South has ever produced. He gave this lady eighty grains of quinine in a few hours. She recovered. The next day she was blind. This was 35 years ago, and the lady still lives, but the amaurosis remains just the same. The beauty, accomplishments, high social position, and wealth of this lovely woman made her case more extensively known than if she had belonged to the humble walks of life. And her case was doubtless influential in aiding Dr. Baldwin's reform in and about Montgomery.

A friend of mine said to me the other day, "Mrs. X. was profoundly malarialized. I gave her 160 grains of quinine in two days, and sent her off to Europe." I am sure this practice is attended with risk, and I think physicians at the North after this hobby has been ridden a while longer as they did at the South, return to more moderate dosing.

I resided in Montgomery when Dr. Baldwin undertook his experiments on dogs as he has described. I assisted him with many of them, and I shall never forget the impression they made on my mind. I had not gone quite mad like some of my friends on giving immense doses of quinine. Still I was giving it in larger doses than I ever gave after seeing Dr. Baldwin's experiments.—*J. Marion Sims, M.D., in Medical Gazette.*

---

THE MONEY QUESTION.—My idea is that the nearer the doctor comes to doing something which is apparent to the patient the more cheerfully will he get his fee. You published something of this sort a few years since in your maxims of success, one of which, if I remember rightly, ran, "Nine tenths of the world employ a doctor to give them physic. Unmixed advice has a doubtful market value." It is a fact. Hygienic medicine is all very good in its way; but it is certainly more profitable for the doctor to open a man's bowels than to throw up his window, and to give him a sole-stirring emetic rather than to shut down on his whisky. I know a somewhat popular, and, I think, a tolerably wise doctor, who tells me that he ranks the compound cathartic pill as the most valuable preparation in the pharmacopœia. Four to six of these form the ordinary dose he prescribes. "Upon my second visit," he says, "of course I find my patient somewhat rattled by his remedy, but all the more does he give me credit for a correct prophecy as to its action; and as he recovers from his prostration in the righting of nature he naturally thinks it was I that cured him "

Perhaps the morals of this sort of practice are a little strained; but I tell you, Mr. Editor, I am a somewhat old-fashioned doctor, and I don't think we are apt to purge too often now a-days, and I am quite certain we don't vomit enough. I take down my father's old account book, and as I scan its long and narrow leaves I see no items—he specified what he did—recorded in his true blue ink oftenest are the prices for a "puke" or a "purge." Well, he was an old country doctor, to be sure; but I tell you he made enough to keep his large family pretty comfortable, and to give me a three-years' keep when I started in city life. A rather extensive neighborhood, too, thought much more of him than it did of Sydenham.

I am a city doctor, though, and I have studied the ways of big practitioners who make their livings among metropolitan patients. Don't you believe it, Mr. Editor, that human nature changes one whit in regard to its notions of disease, among high and low, rich or poor, townspeople or country-folk. With every man and woman of them disease is an *entity*, which must be expelled by direct attack. Fol-de-rol with your popular physiologist. You cannot educate them out of it, and what would be the good if we could? Confidentially, Mr. Editor, suppose we made them such sceptics as some of us, where would our living be from physic?

Well, as I said, I have studied the ways of the city magnates, and I tell you that they do best who prescribe most. I knew a superb doctor. He did the biggest practice in physic that any man did in my city. He had a score of good qualities to be sure. He was intelligent, industrious—he was prompt, attentive (heavens! he *was* a visitor); but I do believe no one of these contributed more to his power than his prescriptions. You never heard him say, "You don't need anything; you will be all right in a day or so." It was, "Take this"——; and, besides leaving a good fortune when he died, he brought comfort to thousands while living.—*Louisville Med. News—Obstetric Gazette.*

---

Bell's Paralysis.—The fact that only the muscles of expression are affected is sufficient to diagnose this form of paralysis from that due to hemiplegia, cerebral hemorrhage, and other causes. The lesion here is evidently in some part of the course of the seventh cranial nerve. The exposure to the cold draught probably set up a rheumatic inflammation of the nerve, which caused an effusion of coagulable lymph within the nerve sheath, which was followed by swelling, and hence pressure on some portion of the temporal bone. This pressure persisting, gave rise to the pain and the paralysis of the nerve. Long continued pressure on any nerve will cause paralysis of the parts supplied by it, as may be evidenced by sitting for some time with the arm hanging over the back of a chair, thus compressing the axillary nerves. When the facial nerve is paralyzed the eye affected cannot wink automatically, hence particles of dust collect on the conjunctiva, and the eye becomes inflamed, and tears form and trickle down the face. This inconvenience may be avoided by keeping the eye closed with a small piece of court plaster, when the patient goes out in the wind. This, also, has a slightly curative influence.

As to treatment, not one case in a thousand can be cured without the use of electricity in some form. The indications are to produce contraction of the paralyzed muscles. If tried early, the faradic current is very efficacious in accomplishing this end. In applying it, place one of the wet sponges of the machine just under the ear on the affected side, and touch all the paralyzed muscles in succession with the other sponge. This should be repeated every day until the patient gains some voluntary power over the muscles. With sittings of about fifteen minutes a day this result will generally be attained within two or three weeks. After this, the application need only be made every other day, for a couple of months, or until perfect control over the muscle is restored. Another very beneficial procedure is, to put a hook made for the purpose, into the angle of the mouth on the paralyzed side, and attach it by the other end to the ear, so as to draw back the relaxed muscles and keep them contracted. Such an apparatus may easily be made, by softening a piece of whalebone in a flame, when it may be easily bent into a hook of the required shape, and then should be polished smooth. It must be long enough to reach from the angle of the mouth to the back of the ear, around which it is retained by another bend Or a simplar modification may be made from a lady's cloak-hook, to which is attached a small rubber band, which encirles the ear. This should be worn at night, and it will be found quite efficacious; often shortening the duration of the disease one half. A cure will frequently be affected by the use of mild galvanism, together with the hypodermic injection of strychnia. The solution may be prepared as follows: To one grain of sulphate of strychnia, add one ounce of water. Of this, inject ten drops the first day, into the face or calf of the leg, or better still into the deltoid muscles. Increase the amount by one drop each day. It is rarely necessary to carry it beyond twenty or twenty-five drops. If the patient is seen while there is still some rheumatic effusion in the sheath of the nerve, blistering or cauterizing over the inflamed portion is often beneficial. By some of these means, about ninety-nine one-hundredths of the simple cases may be cured. If these measures are unsuccessful it is probably because the patient was seen too late.—*Clinical lecture by W. A. Hammond M.D., in Medical and Surgical Reporter.*

Treatment of Heart Diseases.—An interesting review of an article on this subject, in the *Italian Medical Gazette* of January, 1881, appears in the Lyon *Medical* of July 10, 1881. The writer of the article (Prof. Renzi) has evidently studied with care the actions of three important drugs largely used now-a-days in cases af heart disease—viz., bromide of potassium, iodide of potassium, and chloral hydrate; and he has given some important information regarding them. Bromide of potassium is shown to have such a direct influence on the heart and capillaries as to entitle it to a high position among the cardio-vascular drugs. According to Prof. Dujardin-Beaumetz, who considers it one of the best heart tonics we possess, the bromide, besides being a nervine sedative, acts directly on the heart, and lessens considerably any irregular action of that organ. He says that, as a nervine sedative, the drug is useful in counteracting the sleeplessness which so greatly enfeebles and wears out patients suffering from heart disease, while its value in such cases is greatly enhanced by its direct beneficial action on the diseased organ itself. According to Prof. See (largely quoted along with Dujardin-Beaumetz by the writer of the article) bromide of potassium is especially useful in heart affections where we have diminished arterial pressure, rapid and irregular action of the heart, passive congestion, œdema, cyanosis, dyspnœa, and sleeplessness.

Iodide of potassium is shown to be very beneficial in dyspnœa arising from heart disease. It is also of great benefit in arresting degenerative changes in the heart tissue.

The action of chloral hydrate on the heart, as observed by Prof. Renzi, is at once to diminish the rapidity of its action, and after a time to reduce its energy. The drug seems to act on the heart by paralyzing either the cardiac ganglia or the vasculo-motor centres in the brain. The researches of Claude Bernard, Rokitanski, and others, would indicate that the latter are chiefly affected by the administration of chloral, for they found that it caused great diminution of blood pressure by dilatation of the capillaries.

In summing up his observations on the three drugs referred to, Prof. Renzi says of bromide of potassium that it lessens the anxiety of patients suffering from heart disease, gives them a certain sense of comfort, and enables them to breathe freely. Under its influence sleep is more easily obtained, is more tranquil, and of longer duration than when obtained by other drugs. It is, moreover, a more natural sleep. The bromide reduces undue rapidity of the heart's action and of respiration. Cough, however, seems to be aggravated by the use of bromide of potassium alone.

Of iodide of potassium, he says that it is a most useful drug in diseases of the heart. One of its chief effects is a complete relief from dyspnœa and all asthmatic symptoms.

Chloral hydrate is not much esteemed by him. It can procure sleep of a kind, but is of no use in relieving the dyspnœa so troublesome in cases of heart disease. It is, moreover, dangerous when given in conjunction with iodide of potassium, the latter drug apparently having the effect of greatly increasing its soporific action.

From Prof. Renzi's summing up, it would seem that a combination of the iodide and bromide of potassium is a most beneficial remedy in cases of heart disease.—*Med. and Surg. Reporter.*

---

The Action of Mercury in Syphilis.—Weighing all the foregoing reflections together, do we not gain some intimation of the action of mercury in syphilis? The chief characteristic of syphilis is a tendency to hyperplastic cell-growth. In every lesion of the disease, properly so-called, this is the cardinal factor. The effect of mercury is to check the hyperplasia. It does this by imparting to the cells some quality which restrains their morbid activity, and predisposes them to retrograde metamorphosis. It does not act by increasing waste, by denutrition, but by retarding growth; nor does it aid repair. The mercurialized cell performs its vital movements with lessened alacrity; its tendency to respond to the irritating principle of the disease is diminished. Thus, we see, that mercury given before the secondary manifestations appear delays their appearance and often moderates their intensity.—Kussmaul states that he has never known an instance of a worker in quicksilver *acquiring* syphilis, either primary or secondary, so long as he was actually suffering from mercurialism. But, to obtain this inhibitory action of mercury, the organism must be well charged. To this end, it is not large doses that are required, but absorbable ones. The drug must be given in the form in which the largest proportion of mercury will be appropriated by and assimilated with the albuminoid elements of the body. All else is simply waste. Nor is it necessary to induce toxic effects. Salivation may show that the blood is surcharged, but not that the organism is saturated. When each cell and fibre has received its quota of mercury, when all the tissues have been mercurialized, the body is in a state to resist the syphilitic action. But, since elimination is going on constantly, in order to maintain this state of resistance, the treatment must be more or less prolonged. For the remedy does not reach the cause—the active principle of syphilis—it tends only to annul its effects But there comes a time when tissues so abnormally constituted no longer return to a condition of health. The prolonged disturbance of the normal vital movements is followed by a permanent derangement of nutrition—a true cachexia. Compared with the dangers of a grave or malignant form of syphilis, the dangers that attend the prolonged use of mercury may be insignificant. There are many cases of syphilis, however, where we cannot afford to entirely ignore these lesser dangers of the cure, which may, possibly, become worse than those of the disease.—*Edward B. Bronson, M.D., in N. Y. Med. Record.*

---

THE INDISCRIMINATE USE OF QUININE —No remedy of our armamentarium is more abused, or more indiscriminately administered, than the alkaloid from cinchona bark. And this abuse is alarmingly on the increase. It is time the medical men of our country were awaking to the fact; but instead of remedying the evil, we find they either lead the public or the public lead them to giving quinine for every remedy that flesh is heir to.

Dr. S. P. Moore, Surgeon General Confederate Army, positively interdicted by special order the dispensing of quinine to patients other than those suffering from malarial fevers. This order was promulgated at a time when this remedy was very scarce in the Confederacy, and possibly had its origin in this fact; but it is easy to conceive that this accomplished and skillful surgeon and physician, who had sojourned in every part of the world, and who had been a careful observer of disease for thirty years, should have become imbued with the idea that quinine was a remedy as potent for evil as for good—that it was a two-edged sword.

Quinine congests the brain and its meninges, producing dimness of vision, perverting intellection, inducing unsteadiness of gait, ringing of the ears, and often permanent deafness has resulted from its action upon the auditory nerve. Blindness, partial or complete, has occasionally come under my observation. How could all this long train of evils occur without greatly disturbing the entire sensorium by congestion. In large doses, from thirty to sixty grains, it lowers temperature in very much the same way as if a patient had received a sharp blow upon the head. In other words, the impression of large doses of quinine closely resembles the concussion of brain resulting from a blow or fall.

It is my misfortune to frequently see the gravest results from the heroic and indiscriminate use of quinine, particularly in children during the period of dentition. In these cases, with a sensorium already disturbed, it is easy to see what bad results may supervene—such as convulsions, which are often witnessed. Adults as well as infants and children are often so severely impressed with this remedy as to forbid the possibility of their ultimate recovery.

In this country nearly every physician regards irregular febrile movement as evidence of malarial complication. There was never a greater fallacy, and the prevalence of this view has done, and is still doing, an immense amount of mischief.—*W. M. Fuqua, M.D., in Louisville Med. News.*

---

Milk Indigestion in Young Children.—Dr. Eustace Smith, in an article on this subject in the *British Medical*

*Journal*, says that when indigestion is due to catarrh of the stomach it is readily amenable to treatment.

In cases of gastric catarrh, when the complaint is acute and severe, vomiting is usually the most prominent symptom. Under such circumstances milk becomes a positive poison, and no hope of alleviating the symptoms can be entertained while this diet is persisted with. In the case of an infant two months old, brought up by hand, and fed upon milk and barley-water, uncontrollable vomiting and diarrhœa had reduced it to the last extremity. Dr Smith directed a weak mustard poultice to the epigastrium. The milk was stopped, and the child fed with weak veal-broth and thin barley-water, mixed together in equal proportions, and given cold at intervals with a teaspoon. A few drops of brandy were given occasionally, as seemed desirable. As a result of this treatment the vomiting stopped at once, and the child, when seen three days afterwards, was found to be much improved, and was cured by the end of a few days' further treatment. The most important part of the treatment in this case was the substitution of veal broth for milk. Directly the supply of fermentable matter was stopped, fermentation ceased, acid was no longer formed, and the digestive organs returned to a healthy condition. Here the derangement was acute.—*Canada Medical Record.*

An infant two months old, fed on milk and barley-water suffered from indigestion, and, according to the reporter, the milk was the cause. He does not inform us what kind of milk was used, or how it was prepared, except that it was mixed with barley-water.

Can an infant thrive on veal broth and barley-water? Does this compound contain all of the elements necessary for its nutrition? Some remarkable doctrines on the subject of infant feeding are held and practiced. Nature, in this matter, in a safe guide.

---

Ether and Chloroform.—In a recent discussion in the Philadelphia County Medical Society, Prof. Bartholow said he did not believe ether to be necessarily safer than chloroform, but that more deaths from ether may be anticipated when it "comes into more general use." We are rather surprised at this statement, as it implies that ether has not yet had a fair comparative trial. That it has not been employed with nearly so great frequency as chloroform cannot be denied. Nevertheless there are many large cities and extensive regions of country on both sides of the Atlantic where it is used, and has been used for many years, almost exclusively, affording data quite sufficient for positive conclusions in regard to its safety. If there is any single fact beyond controversy, it is that chloroform is more dangerous than ether. Even if it be claimed that chloroform has been employed over ether in the proportion of 100 to 1, the number of deaths reported from it far exceeds that proportion. We notice that in the discussion referred to, the idea is expressed that if ether was administered as carelessly as chloroform, its fatalities would be increased. But is it not certain that chloroform is used with much the greater degree of care? So much confidence have the advocates of ether as to its safety that they are in the habit of giving it lavishly and even recklessly. We have no question that if no greater care and apprehension were associated with chloroform than with ether, casualties from the use of the former would be largely increased. —*Pacific Med. and Surg. Jour.—St. Louis Med. and Surg. Jour.*

---

THE ERA OF "PADS."—The *New York Times* thus pleasantly touches up one of the most successful, though most transparent, of the humbugs of the day:

"Since the original liver-pad inventor made a fortune by decorating the fences of our country with his half length portrait, representing him as dressed almost exclusively in a liver-pad, the attention of inventors of remedies for all sorts of diseases has been turned to the exterior of their fellow-beings. Pads have to a great extent superseded 'bitters' and 'compound extracts' and 'favorite remedies,' and the inexhaustible supply of eminent though anonymous ministers of the gospel, who were formerly constantly testifying that they had been cured of incurable diseases after taking seven bottles of this of that medicine, are now devoting most of their spare time to writing letters declaring the miracles wrought in the condition of their various organs by wearing appropriate pads. There is not an organ in the possession of any human being which is without its special pad, and persons who fancy themselves afflicted with a complication of diseases frequently cover themselves so thoroughly with remedial pads that they could with entire decency appear in public without any of the garments usually held to be necessary."—*Boston Journal of Chemistry.*

---

A Breach of the Code.—Among the current literary items is one stating that a number of leading surgeons will present their views of the treatment of the late President's wounds in the December number of the North American Review. The names of these distinguished writers have not transpired, but their distinction is a warranty for the assertion that no one knows better than they that they are about to violate the spirit, if not the letter, of the medical code. Leading surgeons are not usually the last persons to draw attention to the ethical improprieties of less noted brethren, and we see no reason in their eminent position for our withholding judgment upon an act so unbecoming as that now contemplated. No principle of professional ethics is better founded than that which declares it unworthy for a doctor to air his technical learning before the public in a matter not connected with state medicine. For every scientific controversy the medical, not the secular, press is the proper arena. To seek a different public is to show another intent than that of advancing medical science—an intent which will doubtless receive general disapprobation.—*Louisville Med. News.*

Such a course is a breach of right and honesty, as well as of the code. The code and honesty may be always found pointing the same way.

---

The Treatment of Nasal Polypi by Injection.—I have tried pure commercial acetic acid. I have tried it diluted; I have tried the saturated solution of corrosive sublimate in alcohol, and all the other substances already named, and I have arrived at the conclusion that mucoid polyps are best relieved by carbolic acid injections; that the recurrent polyps with fibrous pedicle which may have resisted attempts at removal with forceps, or the action of the galvano-cautery, may, as a general thing, be best removed by injecting either the officinal tincture of iodine or the corrosive sublimate mixture. The myxomatous type of polypus may be removed with chloride of zinc, or a strong solution of corrosive sublimate and muriate of ammonia. The so-called hypertrophied tissue, or redundant mucous membrane covering the turbinated bones, can be made to shrink promptly and efficiently by the injection of the officinal tincture of iodine, which leaves no mark behind, and is attended by but little pain, and this always of temporary duration.

No matter what method is practiced, after-treatment is of more importance than the removal of the polypus itself; because, unless the rhinitis which always attends the presence of polypoid growths of the nose—and which never ceases by their mere removal—is controlled, it will undoubtedly furnish all the conditions necessary for the reproduction of the growths.—*Dudley Reynolds, M.D., in New York Med. Record.*

---

A Tympanitic Medical School.—The "biggest thing" we know of in the way of modern progress is the "Reform Medical College of Georgia," which proclaims itself "the only Reform Medical College in the world except it may be one in Ohio." It promises to manufacture a Reformed Doctor (qu.? Buchanan) in eighteen months including two short courses of lectures. The faculty has six professors, one of whom resides in Atlanta, two in Macon, two in New York, and one in Michigan. Referring to scholarship tickets, the announcement says: "This will entitle the holder to attend as many courses as they please." It also calls on the young men of Georgia to "Remember that while the profession is full to overflowing of old-school practitioners, there is plenty of room for Reformers, especially up stairs." A bad case of tympanites, we should say.—*Pacific Med. and Surg. Journal.*

Maternal Impressions.—The following occurred in the practice of a Maryland physician, according to the *Dublin Medical Journal:* "A lady during pregnancy carried with her a pocket-edition of Moore's poetical works, which she read almost constantly. Her child, at three years of age, exhibited a most wonderful gift of putting sentences into rhyme; in fact, naturally expressed his little ideas and thoughts in flowing verse!" Blame not the bard — but a case like this shows how important is a well-assorted library to a gravid uterus.—*British Med. Jour.—Louisville Med. News.*

How would it do to make doctors, lawyers and preachers on this simple, poet-born plan?

---

Effects of Excision of the Syphilitic Chancre.—M. Mauriac reports (*Gazette des Hopitaux,*) seven carefully recorded cases in which he excised the initial lesion of syphilis. In six, excision was performed at periods varying from four to sixteen or eighteen days after the appearance of the sore. In the seventh case, the initial lesion was excised about fifty hours after it had been first noticed, and before there was the least trace of glandular enlargement; but in this, as well as in all the others, the operation was unsuccessful in preventing further development of the disease.—*London Medical Record.—American Specialist*

---

Lively For The Surgeons by One of Them.—To-day, we may say, with the deepest conviction, that the surgeon is responsible for every disturbance that occurs in a wound; that it is his fault if even the slightest reaction or redness is developed in it, or if an amputation is not healed by first intention. He must reproach himself severely if after an operation bagging of pus occurs, and especially if death occurs from pyæmia.—*Volkmann, Int. Med. Congress.*

This thing is being run into the ground.

---

VACCINATION.—We contend that small-pox and cow-pox are not identical:

1. Because cow-pox has never been converted by any means into small-pox.

2. Because, in the light of the attempts made by many careful and truthful experimenters, to produce small-pox by the inoculation of the cow with small-pox virus, the number of failures on the part of such a very large majority of them, together with the numerous positive denials made of the possibility of success, it may be concluded that small-pox virus is never converted into vaccine by transmission through the cow. The results of Gassner, of Ceely, and Thiele and Badcock are not left out of consideration in making this statement.

3. Cow-pox does not become epidemic, nor does it spread by effluvia from the human subject. It is always confined to the point of insertion in the human subject, never producing a general eruption of vaccine vesicles.

4. Structurally considered, the vesicles of vaccination and the pustules of inoculation are very dissimilar. Clinically considered, the course and duration of the two diseases are not unlike, but certainly not identical, having enough points of difference to enable the skilled diagnostician to differentiate them.—*Thomas F. Wood, M.D., in Chicago Med. Jour. and Examiner.*

